# *Thymbra capitata* (L.) Cav. and *Rosmarinus officinalis* (L.) Essential Oils: In Vitro Effects and Toxicity on Swine Spermatozoa

**DOI:** 10.3390/molecules22122162

**Published:** 2017-12-06

**Authors:** Alberto Elmi, Domenico Ventrella, Francesca Barone, Gianfranco Filippini, Stefania Benvenuti, Annamaria Pisi, Maurizio Scozzoli, Maria L. Bacci

**Affiliations:** 1 Department of Veterinary Medical Sciences, University of Bologna, Via Tolara di Sopra 50, 40064 Ozzano dell’Emilia, Italy; alberto.elmi2@unibo.it (A.E.); francesca.barone7@unibo.it (F.B.); marialaura.bacci@unibo.it (M.L.B.); 2 Department of Agricultural Sciences, University of Bologna, Via Fanin 44, 40127 Bologna, Italy; gianfranco.filippini@unibo.it (G.F.); annamaria.pisi@unibo.it (A.P.); 3 Department of Life Sciences, University of Modena and Reggio Emilia, Via Giuseppe Campi 103, 41125 Modena, Italy; stefania.benvenuti@unimore.it; 4 APA-CT S.r.l., via Sacco Nicola 22, 47122 Forlì, Italy; maurizio@apabio.it

**Keywords:** essential oils, *Thymbra capitata*, *Rosmarinus officinalis*, swine spermatozoa, in vitro cytotoxicity

## Abstract

Essential oils possess a variety of biological properties (i.e., antioxidant, antibacterial, and cytotoxic) that could possibly be applied in reproductive medicine, but their effects on spermatozoa are still partially unknown. The aim of the study was to describe the effects of *Thymbra capitata* (L.) Cav. and *Rosmarinus officinalis* (L.) essential oils on the main morpho-functional parameters of swine spermatozoa. Essential oils were preliminary characterized by gas chromatography and added with emulsifiers to facilitate diffusion. Experimental samples were prepared by suspending a fixed number of spermatozoa in 5 mL of medium with 10 different concentrations of essential oil (0.2–2 mg/mL, at intervals of 0.2). After 3 h of incubation, samples were analyzed for pH, viability, objective motility, and acrosome status. Results showed that the effects of the essential oils are concentration-dependent and that *R. officinalis* is well tolerated up to 0.6 mg/mL. *T. capitata* impaired the spermatozoa starting from the lowest concentration, with complete spermicidal effect from 0.4 mg/mL. The patterns of damage, confirmed by SEM, were different and quite distinct. As expected, spermatozoa proved to be sensitive to external stimuli and capable of showing different functional patterns, providing interesting insights to the action/toxicity mechanisms. The results of the present work represent the first step towards the systematic characterization of the effects of these compounds on spermatozoa. This kind of studies are necessary to strengthen the idea of future applications of essential oils in the reproductive field due to their antioxidant, antibacterial, or spermicidal properties.

## 1. Introduction

Looking back in history, it is safe to say that traditional medicine has used plants and their derivatives in order to cure and prevent diseases for centuries. During the last decades, the scientific community, in particular the medical one, has witnessed a considerably growing interest towards the application of essential oils (EOs) that are the products of the secondary metabolism of aromatic plants represented by complex mixtures of several compounds, including terpenoids and phenylpropanoids [1,2]. Essential oils and their constituents are indeed effective against a large variety of organisms, including bacteria [3,4], viruses [5,6], and fungi [7]. Moreover, some constitutive components of EOs have been proven to have important antioxidant effects [8]. Nonetheless, it has been acknowledged that those substances show strong toxic activities when applied to different cell populations, including fibroblasts and epithelial cells, monocytes, neutrophils, and also spermatozoa [1,9,10], however without specific cellular targets [8,11].

All the above-mentioned properties, both positive and negative, initiate an extremely interesting discussion related with their application in the reproductive field, both human and veterinary. The toxic effects are probably more investigated and exploited in the human reproductive area, where spermicidal medical devices are constantly being designed [1,10,12]. On the other hand, molecules with proven antibacterial and antioxidant properties are necessary in veterinary artificial insemination, where refrigeration of the inseminating doses represents a zootechnical routine, for example in swine. This fact stems from the necessity to find alternatives to the use of antibiotics that is increasingly being limited by the European Commission [13] and to find molecules capable of ensuring improved quality and high fertility of the male gametes. 

Spermatic cells represent a good model for in vitro toxicological evaluation of several compounds that can affect the reproductive process such as smoke, nicotine [14,15], and other substances [16]. The use of spermatozoa collected from humans unfortunately has several ethical and legislative limitations, thus, animals’ sperm is often used as a translational model. As opposed to macaques, which are probably considered as the overall “gold standard” animal models, porcine spermatozoa are collected using non-invasive techniques (e.g., hand-gloved technique) from trained animals that do not require any sedation and provide high-quality samples [17]. To date, only few EOs, including *Trachyspermum ammi* [10] and *Thymus mumbianus* [1], have been tested on human spermatozoa showing spermicidal capabilities. The mechanism of actions of these compounds are still partially unknown and potentially extremely variable, thus, specific studies for each EO on different cell models are necessary. Concerning animal spermatozoa, in particular porcine ones, only preliminary data regarding the effects of a combination of two EOs has been reported [18]. 

The aim of the present study was to assess and describe the effects of *Thymbra capitata* (L.) Cav. [=*Coridothymus capitatus* (L.) Rchb.f., *Thymus capitatus* Hoff et Link.] and *Rosmarinus officinalis* (L.) essential oils on the main morpho-functional parameters of swine spermatozoa. These plants, belonging to the Lamiaceae family, are endemic and extremely common to the Mediterranean Basin and are indeed proven to show a variety of biological activities [19,20] with possible applications in zootechnical and reproductive medicine. Moreover, preliminary toxicological screening tests on spermatozoa may provide interesting information regarding the mechanisms of action of these compounds.

## 2. Results

### 2.1. Chemical Composition of the EOs

The chemical composition of *T. capitata* (*Tc*) and *R. officinalis* (*Ro*) EOs used in the present study are summarized in Table 1 and Table 2, respectively. Carvacrol was the main component of *Tc* EO (65.2%), followed by p-Cymene (12.28%) and γ-terpinene (5.62%). This particular composition resembles what already described in literature [21].

Regarding *Ro* EO, α-pinene (23.55%), camphor (22.03%), and 1.8-cineole (21.36%) were almost equally represented, followed by camphene (10.16%) and β-pinene (5.39%). When compared to the analyses of *Ro* EOs in the literature, the one used in this study seems to be similar with the exception of the relatively higher quantity of camphor [19].

### 2.2. Semen Morpho-Functional Evaluations

The descriptive statistics regarding the effects of the two tested essential oils on the main morpho-functional characteristics of semen are reported in Table S1 (*Tc*) and S2 (*Ro*).

The ANOVA outputs showed that the effects of both EOs were statistically significant with regard to sperm viability (V) (*Tc p* < 0.0001; *Ro p* < 0.0001), total motility (TotM) (*Tc p* < 0.0001; *Ro p* < 0.0001), progressive motility (ProgM) (*Tc p* < 0.0001; *Ro p* < 0.0001), and acrosome reaction (AR) (*Tc p* < 0.0001; *Ro p* = 0.0036). The analyses of variance for pH, for both EOs, did not show any difference (*Tc p* = 0.9966; *Ro p* = 0.9999).

**Figure 1:**
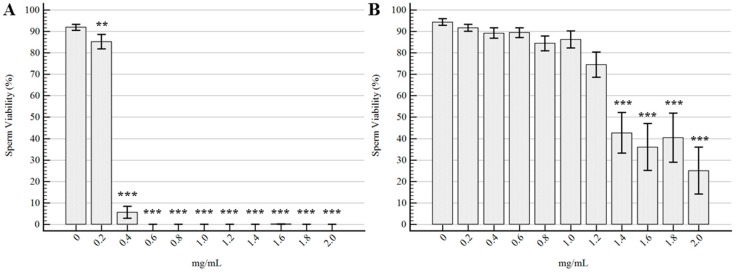
Effects of the EOs on sperm viability. (**A**) *Thymbra capitata*; (**B**) *Rosmarinus officinalis*. Data are expressed as mean ± standard error of the mean (*n* = 6). 0 mg/mL represents the control sample (only emulsifiers). ** = *p* < 0.01; *** = *p* < 0.001.

The effects on sperm viability of the different concentrations of EOs compared to the control samples are represented in Figure 1. *Tc* EO determined a significant reduction in V starting from the lowest tested concentration of 0.2 mg/mL (*p* = 0.005), with stronger effects from 0.4 mg/mL up to 2 mg/mL (*p* < 0.0001). No significant differences were detected for V of spermatozoa treated with *Ro* EO at concentrations ranging from 0.2 mg/mL to 1.2 mg/mL, even if a decreasing trend was observed. Starting from 1.4 mg/mL of *Ro* EO, the effect on V was statistically evident (*p* < 0.001).

**Figure 2:**
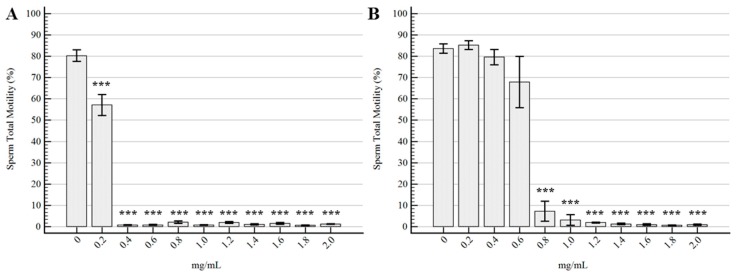
Effects of the EOs on total motility. (**A**) *Thymbra capitata*. (**B**) *Rosmarinus officinalis*. Data are expressed as the mean ± standard error of the mean (*n* = 6), and 0 mg/mL represents the control sample (only emulsifiers). *** = *p* < 0.001.

The trends of total motility for the different samples are represented in Figure 2. The *Tc* EO determined a strong reduction in total motility at 0.2 mg/mL, with an almost complete immobilization of spermatozoa at the other tested concentrations. On the other hand, *Ro* EO did not determine any significant difference in comparison to the control samples up to 0.6 mg/mL, although the concentration of 0.6 mg/mL showed a mild decreasing trend (*p* = 0.08).

The statistical results for ProgM (Figure S1) showed exactly the same trends and differences of TotM for both EOs. The results of the analyses of the kinematic parameters of the samples with a TotM ≥ 20% are summarized in Table S3. It was not possible to accurately analyze the kinematic parameters of the other samples (from 0.4 mg/mL of *Tc* EO; from 0.8 mg/mL of *Ro* EO) because of the extremely low number of motile cells. The velocity (velocity average path: VAP; velocity curved line: VCL; velocity straight line: VSL) and distance (distance average path: DAP; distance curved line: DCL; distance straight line: DSL) parameters showed the same behavior as TotM, with statistical differences starting from 0.2 mg/mL for *Tc* EO. Linearity (LIN), straightness (STR), and wobble (WOB) percentages, alongside with the amplitude of lateral head displacement (ALH) and the beat cross frequency (BCF), showed no significant differences among the analyzed samples.

**Figure 3:**
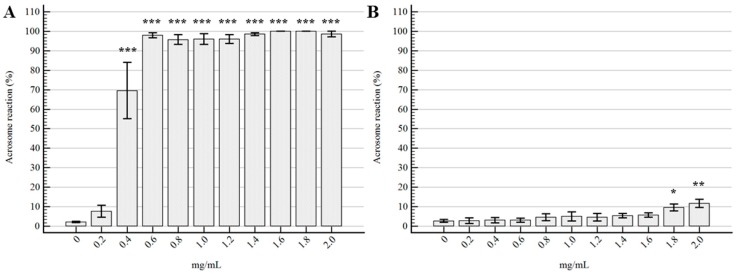
Effects of the EOs on acrosome status. (**A**) *Thymbra capitata*; (**B**) *Rosmarinus officinalis*. Data are expressed as mean ± standard error of the mean (*n* = 6). 0 mg/mL represents the control sample (only emulsifiers). * = *p* < 0.05; ** = *p* < 0.01; *** = *p* < 0.001.

Regarding the percentage of acrosome reaction, the results and statistical differences are represented in Figure 3: *Tc* EO, at the concentration of 0.2 mg/mL did not determine any difference compared to the control sample (*p* = 0.93), whereas all the other experimental concentrations showed important differences (*p* < 0.0001). On the other hand, it was evident that the effect of *Ro* EO on AR was significantly different only for 1.8 and 2 mg/mL, with respective *p*-values of 0.004 and 0.0003.

The angular coefficients (β) resulting from the simple linear regression models between EO concentrations and semen morpho-functional parameters are reported in Table 3.

### 2.3. Morphological Evaluation by SEM

**Figure 4:**
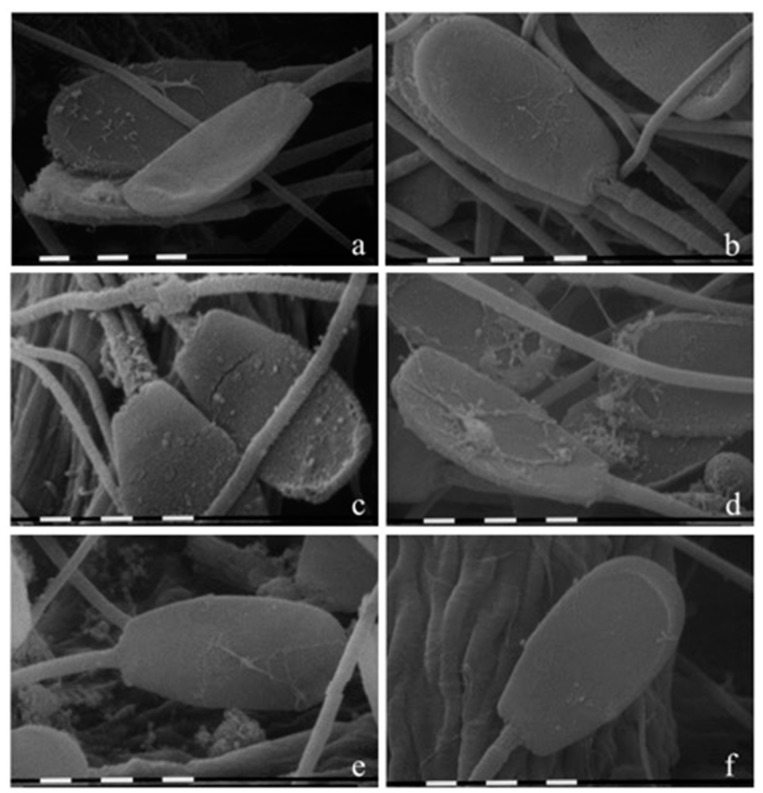
Scanning electron micrographs on the effect of EOs on sperm morphology. Semen samples (**a**) treated with 0.2 mg/mL of *Thymbra capitata*; (**b**) with 0.2 mg/mL of *Rosmarinus officinalis*; (**c**) with 2 mg/mL of *Tc*; (**d**) with 2 mg/mL of *Ro*; (**e**) capacitated spermatozoa; and (**f**) control sample (bars = 1 µm).

Scanning electron microscopy analyses highlighted great alterations of EO-treated spermatozoa compared to both the control sample and the additional capacitated sample, as shown in Figure 4.

As expected, the control (Figure 4f) did not appear morphologically altered: the membranes were regular, smooth, and flattened, and every part of the cell was clearly distinguishable. The capacitated sample (Figure 4e) showed mild exfoliation and disruption of the head membranes in agreement with human literature [22]. On the other hand, the spermatozoa treated with the highest concentration of both EOs showed several morphology defects on the sperm membranes. As shown in Figure 4c, *Tc* (2 mg/mL) caused vesiculation, vacuolation, and lyses of membranes throughout the entire cell. At the same concentration, *Ro* (Figure 4d) only seemed to alter the head region with a different morphological aspect. Figure 4a,b report the spermatozoa treated with 0.2 mg/mL of *Tc* and *Ro*, respectively. In this case, the morphology did not seem to differ compared to the control spermatozoa.

## 3. Discussion

In the present study, different concentrations of *T. capitata* and *R. officinalis* EOs were applied to swine spermatozoa to investigate the effects on their main morpho-functional parameters. The rationale behind the work was the idea of better understanding the interactions between essential oils and male gametes in the light of the increasing interest toward natural substances and their future applications in veterinary and human reproductive medicine. Our data show clear concentration-dependent effects for both tested essential oils as confirmed by the simple linear regression models. 

The results suggest that the essential oil derived from *T. capitata* induce more intense effects, even at the lowest tested concentration of 0.2 mg/mL. Viability and motility are, indeed, significantly reduced, resulting in semen deterioration below the accepted quality standards. At the same concentration, the percentage of spermatozoa with reacted acrosomes show an increasing trend, but in a non-statistically significant manner. Starting at the concentration of 0.6 mg/mL, this EO induces a complete spermicidal effect, completely altering all the investigated parameters. The *Tc* EO used in this study is composed by carvacrol (65.2%) and thymol (3.49%), compounds that comprise the majority of its phenolic content. It has already been reported that the terpene phenols, in particular carvacrol and thymol, are the major compounds responsible for the antimicrobial effects of EOs [23]. They are, indeed, capable of altering the permeability of bacterial membranes by joining to the amine and hydroxylamine groups of the membrane proteins [20]. Our hypothesis is that such a high content in phenols of this particular EO has caused alterations in the spermatozoa membrane similar to that described in bacteria. This statement seems to be strongly supported by the findings regarding the viability and acrosome status. Spermatozoa viability, when assessed with the eosin-nigrosin staining technique, is actually a measure of the cytoplasmic membrane integrity [24] since the eosin only penetrates the head of spermatozoa with disrupted outer membranes. Therefore, this parameter can be considered as an indirect index of membrane deterioration. Likewise, the staining technique used to assess the percentage of the reacted acrosome, Comassie Blue, gives us information regarding the status of the acrosomal membranes. In the case of *Tc* EO, as previously reported, these two parameters were drastically affected, suggesting an extremely high level of membrane alterations. The above hypothesis seems to find a final iconographic validation in the SEM images, especially for the sample treated with 2 mg/mL, which display severe outer alterations on the entire cell. In the light of the results achieved by the present study, *T. capitata* seems to have a greater potential as a spermicidal agent for reproductive medicine. Nonetheless, analyses on spermatozoa treated with lower concentrations are necessary to identify a potentially harmless concentration.

As far as it concerns the analyses regarding the *R. officinalis* EO, the results are clearly different. Overall, this compound seems to be well tolerated by boar spermatozoa up to the concentration of 0.6 mg/mL. The membrane integrity, in terms of viability and acrosome reaction, is preserved up to concentrations of 1.2 mg/mL and 1.6 mg/mL respectively, never reaching an absolute spermicidal effect. In this case, the limiting parameter seems to be the motility, both total and progressive, which appears significantly altered starting from the concentration of 0.8 mg/mL. The inhibition of motility without any structural membrane alteration, represents a different pattern of damage when compared to the one induced by the *Tc* EO, and proves why the motility is one of the most important and sensitive parameter when analyzing spermatozoa [25] and spermicidal substances [12]. Bakkali and colleagues have reported that, in eukaryotic cells, EOs can depolarize mitochondrial membranes by decreasing membrane potential and affecting ionic cycling [8], and this might be the reason behind the motility inhibition. Due to its relatively wider “safety window”, the EO of *R. officinalis* may be exploited, in reproductive medicine, for its antioxidant and antibacterial effects. For instance, Chaftar et al. [26] reported that the minimum inhibitory concentration (MIC) of this EO against several strains of Legionella pneumophila is <0.55 mg/mL, a concentration that, according to the present study, would be well tolerated by spermatozoa. Further studies are necessary to confirm its potential as an antibacterial agent, for example, in artificial insemination swine doses, where antibiotics are still mandatory [13].

## 4. Materials and Methods

The EOs of *Tc* and *Ro* used in this study were kindly supplied by APA-CT S.r.l. (Via Sacco Nicola, 22 47122, Forlì, Italy). For the experimental purposes, the EOs were reconstituted in 0.5% dimethylsulfoxide (DMSO) with Tween 80 (0.02% *v*/*v*) for easy diffusion [3].

### 4.1. Chemo-Characterization of the EOs

#### 4.1.1. Gas Chromatography-Mass Detector (GC-MS) Analysis

Analyses were performed on a 7890A gas chromatograph coupled with a 5975C network mass spectrometer (Agilent Technologies, Waldbronn, Germany). Compounds were separated on an Agilent Technologies HP-5 MS cross-linked poly-5% diphenyl-95% dimethyl polysiloxane (30 m × 0.25 mm i.d., 0.25 μm film thickness) capillary column. The column temperature was initially set at 45 °C, then increased at a rate of 2 °C/min up to 100 °C, then raised to 250 °C at a rate of 5 °C/min, and finally held for 5 min. The injection volume was 0.1 μL, with a split ratio 1:20. Helium was used as the carrier gas, at a flow rate of 0.7 mL/min. The injector, transfer line and ion-source temperature was 250 °C, 280 °C, and 230 °C, respectively. MS detection was performed with electron ionization (EI) at 70 eV, operating in the full-scan acquisition mode in the *m*/*z* range 40–400. EOs were diluted 1:20 (*v*/*v*) with n-hexane before GC-MS analysis.

#### 4.1.2. Gas Chromatography-Flame Ionization Detector (GC-FID) Analysis

Analyses were carried out on an Agilent Technologies 7820 gas chromatograph (Waldbronn, Germany) with a flame ionization detector (FID). Compounds were separated on an Agilent Technologies HP-5 crosslinked poly-5% diphenyl-95% dimethyl polysiloxane (30 m × 0.32 mm i.d., 0.25 mm film hickness) capillary column. The temperature program was the same as described above. The injection volume was 0.1 μL in split mode 1:20. Helium was used as the carrier gas at a flow rate of 1.0 mL/min. The injector and detector temperature was set at 250 °C and 300 °C, respectively. EOs and the reference standards were diluted 1:20 (*v*/*v*) with *n*-hexane before GC-FID analysis. The analyses were performed in duplicate.

#### 4.1.3. Qualitative and Semi-Quantitative Analysis

Compounds were identified by comparing the retention times of the chromatographic peaks with those of authentic reference standards run under the same conditions and by comparing the linear retention indices (LRIs) relative to C8–C40 n-alkanes obtained on the HP-5 column under the above-mentioned conditions with the literature [27]. Peak enrichment by co-injection with authentic reference compounds was also carried out. Comparison of the MS-fragmentation pattern of the target analytes with those of pure components was performed. A mass-spectrum database search was carried out by using the National Institute of Standards and Technology (NIST, Gaithersburg, MD, USA) mass-spectral database (version 2.0d, 2005).

Semi-quantification was calculated as the relative percentage amount of each analyte; in particular, the values were expressed as the percentage peak area relative to the total composition of each EO obtained by GC-FID analysis.

### 4.2. Boars and Ejaculate Collection

Two adult hybrid (Large White × Duroc) boars were enrolled as sperm donors for the experiments. Animals were housed in single pens, according to the national law (D.lgs n.122/2011). Semen was routinely collected by an experienced operator twice a week using the hand-gloved technique. The sperm-rich fraction (SRF) was immediately diluted 1:1 *v*/*v* with Swine Fertilization Medium (SFM) extender prepared as previously described [28], and an aliquot (2 mL) was analyzed to assess overall quality [29]. Each SRF was evaluated for spermatozoa concentration by a Thoma haemocytometer chamber, viability and total motility, as described later.

SRF inclusion criteria for the experimental protocol were set as sperm V > 85% and TotM > 80%, according to the common sperm quality standards. Twelve ejaculates, six from each boar, were included in experimental protocol.

### 4.3. Experimental Protocol

Each EO was tested on six different ejaculates (*n* = 6) (three from each boar). The experimental doses were prepared by suspending a fixed number of spermatozoa (15 × 10^7^ spz) in 5 mL of SFM extender (final concentration = 3 × 10^7^ spz/mL) with 10 different concentrations of EO (from 0.2 to 2 mg/mL, at intervals of 0.2). The SFM extender was prepared as described by Fantinati et al. [28], without any antibiotic. For each experiment, control samples were realized by only adding the emulsifiers (DMSO 0.5% *v*/*v* and Tween 80 0.02% *v*/*v* [3]). After preparation, the experimental doses were incubated for 3 h in a refrigerated bath at 16 °C (±1 °C), and subsequently evaluated for the principal morpho-functional parameters.

### 4.4. Semen Morpho-Functional Evaluations

Viability was assessed using the Eosin-Nigrosin staining method [29]. Briefly, 10 µL of staining solution were added to 10 µL of each dose, and 8 µL were immediately smeared on a glass microscope slide for the analysis. The percentage of live cells (undyed spermatozoa/all spermatozoa) was evaluated on a minimum of 200 cells.

Analyses regarding the acrosome status were performed using a modified Coomassie Blue staining protocol as previously described [30]. After two washings in phosphate-buffered saline (PBS), spermatozoa were fixed in 4% paraformaldehyde for 10 min. Sperm were then centrifuged and suspended with ammonium acetate (100 mM, pH 9). Twenty microliters were then smeared on a microscope glass slide, air dried, and incubated for 2 min with 0.22% Coomassie Blue G250 staining solution. The percentage of reacted acrosomes (AR) (undyed acrosomes/all acrosomes) was evaluated on a minimum of 200 cells. The slides prepared for the evaluation of viability and acrosome status were coded and analyzed by a blinded operator in order to avoid biases.

The objective motility of the spermatozoa, both total and progressive, and the kinematics parameters were assessed using a Computer Assisted Sperm Analysis (CASA; Hamilton Thorne CEROS II; Animal Motility II, Software Version 1.9, Beverly, MA, USA) unit. Prior to the analysis, an aliquot from each experimental sample was incubated for 10 min at 37 °C in a digital incubator (INCU-Line IL23; VWR International, Radnor, PA, USA). All samples were analyzed by the same blinded operator, and at least one thousand spermatozoa for each sample were tracked. Since the kinematics parameters derive from motile spermatozoa, we decided to only report the ones for samples with a minimum TotM of 20% (at least 200 motile cells).

The pH of each experimental sample was analyzed using a Medidor PH BASIC 20 (Hach Lange srl, Milan, Italy) after calibration according to the instrument’s instructions.

### 4.5. Scanning Electron Microscopy (SEM)

Scanning electron microscopy (SEM) studies were performed to visualize any possible membrane morphological change in the spermatozoa treated with *Tc* and *Ro* EOs. The samples treated with the highest and lowest concentration of each EO (0.2 and 2 mg/mL), one control with the emulsifiers, and an additional capacitated sample [31], were analyzed with SEM. For each sample, an aliquot of 500 μL was centrifuged at 800× *g* for 10 min, and the pellet fixed in 500 μL of 5% glutaraldehyde solution buffered at pH 7.2 with phosphate buffer 0.1 M. The mixture was dropped by a pipette on filter paper, washed with phosphate buffer 0.1 M pH 7.2, dehydrated through an increasing concentration of aqueous ethanol (10%, 20%, 30%, 50%, 75% and 95%) for 15 min and in 100% ethanol for 5 min. All the above steps were performed at 5 °C. The samples were then dried with an Emitech K850 (Emitech Ltd., Ashford, UK) critical point drier unit mounted on aluminum stubs with double-sided tape and coated with gold-palladium film using an Emitech K500 (Emitech Ltd., Strovolos, Cyprus) ion sputtering unit. Samples were observed with a Philips 515 SEM scanning electron microscope (Philips, Eindhoven, The Netherlands) at 10 kV, and pictures were taken with a Nikon 5400 Coolpix digital camera (Nikon, Tokyo, Japan).

### 4.6. Statistical Methods

The statistical analyses were performed using the software R 3.0.3 (The R Foundation for Statistical Computing). Descriptive statistics of the parameters were calculated and expressed as means and standard error of the mean. Normal distribution was assessed with the Shapiro-Wilk test (*p* < 0.05). To evaluate differences between the control doses and the others containing EOs, one-way ANOVA was performed with the significance level set at 0.05. Post hoc analyses were performed by means of Dunnett’s test (*p* < 0.05) to assess the differences between the controls and the treatments. To evaluate the presence of a concentration-dependent effect of the EOs on the morpho-functional parameters, simple linear regression models were performed.

## 5. Conclusions

In conclusion, the proposed approach to evaluate the effects of the essential oils on spermatozoa seems to be capable of providing robust and repeatable results. As expected, these cells proved to be sensitive and susceptible to external stimuli and to be capable of showing a variety of different functional patterns, providing interesting insights to the toxicity mechanisms. Overall, the results of the present work represent the first step towards the systematic characterization of the effects of these natural compounds on spermatozoa. These kinds of studies are necessary to strengthen the idea of future applications of EOs in the reproductive field for their antioxidant, antibacterial, or spermicidal properties.

## Figures and Tables

**Table 1:** Composition of the essential oil of *Thymbra capitata* (L.) Cav.

Compounds	LRI ^1^	Area %
α-Thujene	927	0.71
α-Pinene	933	0.96
Camphene	948	0.15
β-Pinene	976	0.13
β-Myrcene	993	1.37
α-Phellandrene	1006	0.16
α-Terpinene	1017	1.31
p-Cymene	1026	12.28
Limonene	1029	0.45
γ-Terpinene	1060	5.62
trans-sabinene hydrate	1067	0.09
α-Terpinolene	1089	0.21
Linalool	1102	2.37
Borneol	1167	0.18
Terpinen-4-ol	1179	0.7
Thymol	1296	3.49
Carvacrol	1312	65.2
β-Caryophyllene	1426	1.92
α-Humulene	1456	0.11
Caryophyllene oxide	1594	0.12
Total		97.54

^1^ LRI: Linear Retention Index.

**Table 2:** Composition of the essential oil of *Rosmarinus officinalis* (L.).

Compounds	LRI ^1^	Area %
α-Pinene	936	23.55
Camphene	949	10.16
β-Pinene	977	5.39
β-Myrcene	993	1.88
p-Cymene	1026	2.8
1,8-Cineole	1034	21.36
Linalool	1103	0.96
Camphor	1145	22.03
Borneol	1168	2.84
Terpinen-4-ol	1180	0.05
α-Terpineol	1193	2.45
Bornyl acetate	1290	1.38
β-Caryophyllene	1427	1.19
Caryophyllene oxide	1594	0.17
Total		96.22

^1^ LRI: Linear Retention Index.

**Table 3:** Simple linear regression models’ angular coefficients (β).

Parameters	*T. capitata* EO	*R. officinalis* EO
	β (95% C.I.) *p* Value	β (95% C.I.) *p* Value
V %	−0.013 (−0.017; −0.009) *p* < 0.0001	−0.018 (−0.022; −0.015) *p* < 0.0001
TotM %	−0.015 (−0.020: −0.010) *p* < 0.0001	−0.014 (−0.017; −0.012) *p* < 0.0001
ProgM %	−0.027 (−0.037; −0.016) *p* < 0.0001	−0.026 (−0.031; −0.021) *p* < 0.0001
AR %	0.013 (0.010; 0.016) *p* < 0.0001	0.086 (0.052; 0.119) *p* < 0.0001
pH	0.508 (−1.168; 2.185) *p* = 0.546	−0.018 (−2.275; 2.238) *p* = 0.987

C.I. = confidence interval; V = Viability; TotM = total motility; ProgM = progressive motility; AR = acrosome reaction.

## Supplementary Materials

The following are available online. Table S1: Descriptive statistics of the effects of *Thymbra capitata* EO on semen morpho-functional parameters. Table S2: Descriptive statistics of the effects of *Rosmarinus officinalis* EO on semen morpho-functional parameters. Table S3: Effects of *Thymbra capitata* and *Rosmarinus officinalis* EOs on spermatic kinematic parameters. Figure S1: Effects of the EOs on Progressive Motility.
