# Supplementary material for: Thymbra capitata (L.) Cav. and Rosmarinus officinalis (L.) Essential Oils: In Vitro Effects and Toxicity on Swine Spermatozoa
Source: Molecules. 2017 Dec 6;22(12):2162. doi: 10.3390/molecules22122162 (PMC6149686; doi:10.3390/molecules22122162)
Supplement: Supplementary file 1 [file molecules-22-02162-s001.pdf]

Supplementary materials: *Thymbra capitata* (L.) Cav. and *Rosmarinus officinalis* (L.) Essential Oils: *in vitro* effects and toxicity on swine spermatozoa.

Table S1. Descriptive statistics of the effects of *Thymbra capitata* EO on semen morpho-functional parameters. Data are reported as Mean (standard error of the mean), *n*=6.

	***Thymbra capitata* (mg/mL)**										
	**0**	**0.2**	**0.4**	**0.6**	**0.8**	**1**	**1.2**	**1.4**	**1.6**	**1.8**	**2**
**V** %	91.9 (1.4)	85.1 (3.4)	18.8 (2.8)	0	0	0	0	0	0	0	0
**TotM** %	80.2 (2.7)	57.1 (4.9)	0.7 (0.2)	0.8 (0.2)	2.1 (0.6)	0.8 (0.2)	1.9 (0.5)	1.0 (0.2)	1.4 (0.4)	0.5 (0.2)	1.2 (0.2)
**ProgM** %	46.8 (5.7)	24.0 (5.1)	0.1 (0.1)	0.1 (0.1)	0.1 (0.1)	0	0.1 (0.1)	0.1 (0.1)	0.3 (0.1)	0.1 (0.1)	0.1 (0.1)
**AR** %	2.1 (0.4)	7.6 (3.0)	69.5 (14.5)	97.8 (1.3)	95.7 (2.5)	95.9 (2.7)	96.0 (2.3)	98.5 (0.7)	100 (0.0)	100 (0.0)	98.5 (1.5)
**pH**	6.7 (0.0)	6.7 (0.0)	6.7 (0.0)	6.7 (0.0)	6.7 (0.0)	6.7 (0.0)	6.7 (0.0)	6.7 (0.0)	6.7 (0.0)	6.7 (0.0)	6.7 (0.1)

V= Viability; TotM= Total Motility; ProgM= Progressive Motility; AR= Acrosome Reaction.

Table S2. Descriptive statistics of the effects of *Rosmarinus officinalis* EO on semen morpho-functional parameters. Data are reported as Mean (standard error of the mean), *n*=6.

	***Rosmarinus officinalis* (mg/mL)**										
	**0**	**0.2**	**0.4**	**0.6**	**0.8**	**1**	**1.2**	**1.4**	**1.6**	**1.8**	**2**
**V** %	94.3 (1.5)	91.7 (1.6)	89.2 (2.4)	89.4 (2.2)	84.4 (3.4)	74.0 (4.0)	74.5 (5.9)	42.6 (9.5)	36.0 (11.0)	40.4 (11.4)	25 (11.0)
**TotM** %	83.5 (2.2)	85.1 (2.1)	79.5 (3.6)	67.8 (12.1)	7.2 (4.7)	3.1 (2.5)	1.9 (0.2)	1.2 (0.3)	0.9 (0.4)	0.6 (0.2)	0.9 (0.3)
**ProgM** %	45.4 (5.2)	38.8 (7.0)	36.8 (5.0)	31.1 (7.3)	2.1 (0.9)	0	0.1 (0.1)	0.1 (0.1)	0	0	0.1 (0.0)
**AR** %	2.6 (0.7)	2.8 (1.5)	3 (1.4)	3 (1.1)	4.5 (1.8)	5 (2.3)	4.5 (2.0)	5.3 (1.1)	5.6 (1.2)	9.5 (1.8)	11.6 (2.1)
**pH**	6.7 (0.0)	6.7 (0.0)	6.7 (0.0)	6.7 (0.0)	6.7 (0.0)	6.7 (0.0)	6.7 (0.0)	6.7 (0.0)	6.7 (0.0)	6.7 (0.0)	6.7 (0.0)

V= Viability; TotM= Total Motility; ProgM= Progressive Motility; AR= Acrosome Reaction.

Table S3. Effects of *Thymbra capitata* and *Rosmarinus officinalis* EOs on spermatic kinematic parameters. In the table are only reported the samples with a total motility ≥ 20%. Data are reported as Mean (standard error of the mean), *n*=6. Differences were calculated by means of Dunnett PostHoc test (* = p<0.05; ** = p<0.01; *** = p< 0.001).

	***Tc* (mg/ml)**		***Ro* (mg/mL)**			
	**0**	**0.2**	**0**	**0.2**	**0.4**	**0.6**
**TotM** (%)	80.2	57.1**	83.5	85.1	79.5	67.8
	(2.7)	(4.9)	(2.2)	(2.1)	(3.6)	(12.1)
**VAP** (μm/s)	90.0	48.8**	86.4	77.1	84.0	73.9
	(5.4)	(7.2)	(10.0)	(11.8)	(9.3)	(12.2)
**VCL** (μm/s)	197.4	106.6**	189.0	170.7	189.4	167.7
	(11.3)	(16.5)	(22.7)	(26.5)	(21.3)	(27.1)
**VSL** (μm/s)	49.7	26.7**	47.4	43.2	42.5	37.9
	(4.5)	(3.9)	(4.7)	(6.07)	(4.7)	(5.4)
**DAP** (μm)	52.3	29.3**	49.9	42.0	48.6	43.7
	(2.4)	(4.9)	(5.6)	(6.4)	(5.4)	(6.4)
**DCL** (μm)	117.1	65.7**	111.5	95.6	112.6	101.6
	(5.6)	(11.4)	(13.0)	(15.2)	(12.6)	(14.4)
**DSL** (μm)	27.1	15.2**	25.7	21.8	22.5	21.0
	(2.0)	(2.7)	(2.1)	(2.9)	(2.5)	(2.4)
**LIN** (%)	25.7	25.1	26.2	25.8	23.0	24.3
	(1.7)	(0.6)	(1.4)	(0.9)	(0.7)	(1.0)
**STR** (%)	54.9	52.8	55.4	54.9	50.7	52.8
	(2.5)	(1.6)	(2.2)	(1.4)	(1.2)	(1.8)
**WOB** (%)	45.6	45.8	46.1	45.2	44.2	44.6
	(1.0)	(0.6)	(0.6)	(0.6)	(0.3)	(0.7)
**ALH** (μm)	9.6	7.3	9.6	9.1	9.4	9.1
	(0.5)	(0.9)	(0.8)	(1.1)	(0.7)	(1.0)
**BCF** (Hz)	36.9	36.9	36.0	36.7	35.6	36.4
	(0.5)	(3.4)	(1.0)	(1.4)	(1.4)	(1.8)

TotM= Total Motility; VAP= velocity average path; VCL= velocity curved line; VSL= velocity straight line; DAP= distance average path: DCL= distance curved line; DSL= distance straight line; LIN= linearity (VSL/VCL); STR= straightness (VSL/VAP); WOB=wobble (VAP/VCL); ALH= amplitude of lateral head displacement; BCF= beat cross frequency.

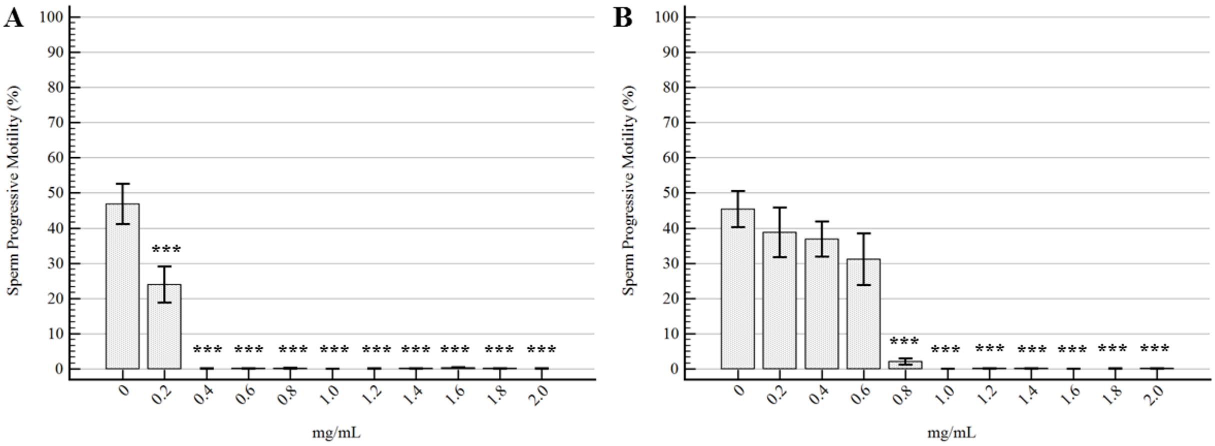

Figure S1. Effects of the EOs on Progressive Motility. (A) *Thymbra capitata*. (B) *Rosmarinus officinalis*. Data are expressed as mean ± standard error of the mean (*n*=6). 0 mg/ml represents the control sample (only emulsifiers). * = $p<0.05$; ** = $p<0.01$; *** = $p< 0.001$.
